# Association of matrix Gla protein polymorphism and knee osteoarthritis in a chinese population

**DOI:** 10.1042/BSR20182228

**Published:** 2019-01-25

**Authors:** Wenpeng Hui, Zhong Cao, Xiao Wang, Junfeng Zhu

**Affiliations:** 1 Department of Spinal Surgery, Shandong Provincial Western Hospital, 4 Duanxing West Road, Jinan, Shandong, China; 2 Department of Orthopedic, Suichang People’s hospital, 143 Miaogao North Street, Suichang, Lishui, Zhejiang, China

**Keywords:** MGP, Osteoarthritis, Polymorphism, single nucleotide polymorphisms

## Abstract

Several studies have explored the association between matrix Gla protein (MGP) gene polymorphism and knee osteoarthritis (OA) risk; however, they obtained conflicting findings. The present study aims to explore the association of MGP gene polymorphism and OA risk in a Chinese Han population. A total of 256 patients with radiographic knee OA and 327 control subjects were recruited in this case–control study. The genotypes of MGP gene rs1800802 polymorphism was determined by standard PCR and restriction fragment length polymorphism (PCR-RLFP). In this case–control study, we observed that MGP gene rs1800802 polymorphism increased the risk of knee OA. Subgroup analyses also found that rs1800802 polymorphism was related to the elevated risk for knee OA among the female, smoker, drinker, and body mass index (BMI) ≥25 kg/m^2^ groups. In conclusion, this study shows that MGP gene rs1800802 polymorphism is associated with increased risk for knee OA in Chinese Han population and the rs1800802 polymorphism may be a diagnostic marker of radiographic knee OA.

## Introduction

Osteoarthritis (OA), is the most prevalent type of arthritis and main reason for the chronic disability of the elderly [1]. According to the WHO, at least 10% of people aged ≥ 60 years old worldwide suffer from OA, characterized by joint stiffness, limitation of movement, progressive loss of articular cartilage, and variable degrees of inflammation [2]. Biochemical, biomechanical, molecular, and morphological changes can be observed in both cells and extracellular matrix of OA patients. The main pathological changes include fibrillation, osteophytes, loss of articular cartilage, and subchondral bone sclerosis. Knee OA is a multifactorial disease, which may result from several factors, such as obesity, joint trauma, increasing aging trend, and excessive physical activity. Besides those environmental and individual factors, genetic component accounts for nearly up to 50% of the risk of OA development [3]. Previous genome-wide association studies suggested that polymorphisms in some genes may affect the development of OA. Therefore, certain gene studies may provide a novel potential insight and treatment strategy for OA development [4].

Matrix Gla protein (MGP) gene is located at 12p12.3, consisting of four exons [5]. MGP is a member of the vitamin K-dependent (VKD) proteins family, requiring vitamin K to confer function. Bone, cartilage, and vascular smooth muscle were found to contain high amounts of MGP [6]. MGP might be involved in the regulation of cartilage calcification in long bones [7,8]. It works as a potential calcification inhibitor of extracellular matrix in cartilage by clearing calcium phosphate. It was reported that the absence of functional MGP might lead to osteochondrosis in animal models [9].

Evidence suggests that a nonsense mutation of MGP polymorphic sites could produce a truncated protein, which could not bind calcium, resulting in cartilage calcification abnormity and extensive vascular calcification [10]. The effect of MGP gene polymorphism on the development of OA was previously explored [11,12]. Nevertheless, not all of them focused on the knee OA but also on other sites and the findings were still far from a consensus. Thus, this case–control study was designed in a Chinese Han population to investigate the potential relationship between MGP gene rs1800802 polymorphism and knee OA risk.

## Materials and methods

### Patients

From June 2014 to April 2018, 256 knee OA patients and 327 healthy controls were selected from the Shandong Provincial Hospital affiliated to Shandong University And Suichang People’s Hospital. The study was approved by the Ethics Committee of the above two hospitals. We obtained written informed consent from all included patients and controls. Knee OA diagnosis was based on the the American College of Rheumatology Clinical Criteria [2], and the severity of OA was evaluated by the Kellgren–Lawrence (K–L) grade. Furthermore, patients with rheumatoid arthritis, ankylosing spondylitis, autoimmune disorders, or any history of trauma were excluded from this study. The control group had no history, signs, or symptoms of arthritis or joint disease, which was recruited from subjects who received regular health examinations at the above two hospitals. The exclusion criteria for controls were: rheumatoid arthritis, knee replacement, and use of corticosteroid or immunosuppressant drugs.

The demographic data and information on all the established risk factors including sex, age, smoking, drinking, body mass index (BMI) and K–L grading were collected from medical records. Alcoholics were defined as someone who consumed alcohol at least once per week for more than 6 months. Individuals who smoked up to 1 year were considered as smokers.

### DNA extraction and genotyping

Blood samples were collected using vacutainer tubes containg EDTA and stored at −20°C until use. Genomic DNA was isolated from whole blood using the QIAamp DNA Blood Mini Kit (Qiagen, Hilden, Germany). The concentration and quality of extracted DNA were measured using Nano Drop 2000. Genotyping was carried out by standard PCR and restriction fragment length polymorphism (PCR-RLFP). PCRs were performed in a 50 μl volume containing 50 ng of DNA template, 5 μl of 10× PCR buffer, 2.0 mM MgCl_2_, 2.5 U of Taq-DNA-polymerase (LifeTechnologies, Inc.), 0.2 mM dNTPs (Sigma Chemical Co.), and 0.2 mM primers (Biosynthesis). The PCR cycling conditions: an initial denaturation period of 5 min at 95°C, followed by 35 cycles of 30 s at 94°C, 30 s at 60°C, and 1 min at 72°C, and with a final extension at 72°C for 10 min. The digested PCR products were subjected to gel electrophoresis and visualized by ethidium bromide staining. To control the quality of genotyping, the PCR-RFLP method was performed without knowing the status of the cases or controls.

### Statistical analysis

We evaluated the differences in demographics variables and genotype frequencies of rs1800802 polymorphism using Student’s *t* test (for continuous variables) or Chi-squared (χ^2^) test (for categorical variables). The associations between genotype of rs1800802 polymorphism and the risk of knee OA were examined by computing the odds ratios (ORs) and 95% confidence intervals (CIs) using logistic regression analysis. The Hardy–Weinberg equilibrium (HWE) was tested to evaluate whether the control group could represent the whole population by a goodness-of-fit χ^2^ test. Statistical significance was assumed at *P*-values at <0.05 level. Above statistical analyses were performed using the SAS software package (ver. 9.1.3; SAS Institute, Cary, NC, U.S.A.).

## Results

### Characteristics of the study population

Table 1 presents the characteristics of 256 knee OA patients and 327 controls included in the present study. The mean ages of OA patients and healthy controls were 58.39 ± 10.21 and 58.75 ± 9.82 years, respectively. No significant differences in sex, smoking, alcoholism, and BMI were found between the two groups (*P*>0.05). According to the radiographic K–L grading system, approximately 57.4% of OA patients had a KL grading of 3 or 4.

**Table 1:** Patient demographics and risk factors in OA

Variable	Cases (*n*=256)	Controls (*n*=327)	*P*
Age (years)	58.39 ± 10.21	58.75 ± 9.82	0.663
Sex			0.733
Female	157 (61.3%)	196 (59.9%)	
Male	99 (38.7%)	131 (40.1%)	
Smoking status			0.172
Nonsmoker	124 (48.4%)	177 (54.1%)	
Smoker	132 (51.6%)	150 (45.9%)	
Alcoholism			0.766
No	130 (50.8%)	162 (49.5%)	
Yes	126 (49.2%)	165 (50.5%)	
BMI	25.62 ± 3.47	25.99 ± 3.40	0.189
K–L grade			
2	109 (42.6%)		
3	87 (34.0%)		
4	60 (23.4%)		

### Association between MGP gene rs1800802 polymorphism and knee OA risk

The genotype distribution and allele frequencies of rs1800802 polymorphism were provided in Table 2. The frequencies of the AA, AG, GG genotypes of this SNP were 54.7, 36.3, and 8.2% in OA patients, and 61.2, 34.3 and 4.3 in healthy controls. In the control groups, MGP rs1800802 polymorphism conformed to the HWE. Our results indicated that GG genotype had higher susceptibility to OA compared with AA genotype (GG vs AA; adjusted OR: 2.14; 95% CI: 1.05–4.36; *P*=0.035). Similarly, GG genotype was associated with a statistically significant increased risk for knee OA in the recessive model (GG vs AG + AA; OR: 2.01; 95% CI: 1.00–4.03; *P*=0.050). Taking the A allele as reference, a significant association was observed between the presence of G allele and a higher risk of developing OA (G vs A; OR: 1.32; 95% CI: 1.01–1.74; *P*=0.043).

**Table 2:** Logistic regression analysis of associations between MGP rs1800802 polymorphism and risk of OA

Genotype	Cases^1^ (*n*=256)		Controls^2^ (*n*=327)		OR (95% CI)	*P*	Adjusted OR (95% CI)^2^	Adjusted *P*
	*n*	%	*n*	%				
AG vs AA	93/140	36.3/54.7	112/200	34.3/61.2	1.19 (0.84–1.68)	0.338	1.19 (0.84–1.68)	0.341
GG vs AA	21/140	8.2/54.7	14/200	4.3/61.2	**2.14 (1.05–4.36)**	0.035	**2.14 (1.05–4.36)**	0.036
AG + GG vs AA	114/140	44.5/54.7	126/200	38.5/61.2	1.29 (0.93–1.80)	0.131	1.29 (0.93–1.80)	0.133
GG vs AG + AA	21/233	8.2/91.0	14/312	4.3/95.4	**2.01 (1.00–4.03)**	0.050	2.01 (1.00–4.03)	0.051
G vs A	135/373	26.4/72.9	140/512	21.4/78.3	**1.32 (1.01–1.74)**	0.043		

Bold values are statistically significant (*P* <0.05).

1 The genotyping was successful in 254 cases and 326 controls.

2 Adjusted for sex and age.

Subgroup analyses were conducted according to sex, age, smoking, alcoholism, and BMI (Table 3). Stratified analysis by sex indicated that GG genotype suffered higher risk of knee OA in females (GG vs AA; OR: 2.43; 95% CI: 1.02–5.75; *P*=0.044) rather than in males. The significant differences were also found to be existed in the comparison of GG genotype versus combined genotype (AA + AG). This significant association also held true for smokers, alcoholics, and subjects with ≥25 kg/m^2^ groups.

**Table 3:** Stratified analyses between MGP rs1800802 polymorphism and the risk of OA

Variable	MGP rs1800802 (case–control)			AG vs AA	GG vs AA	AG + GG vs AA	GG vs AG + AA
	AA	AG	GG				
Sex							
Male	55/84	39/42	5/5	1.42 (0.82,2.47); 0.215	1.53 (0.42,5.52); 0.518	1.43 (0.84,2.44); 0.189	1.34 (0.38,4.76); 0.651
Female	85/116	54/70	16/9	1.05 (0.67,1.65); 0.824	**2.43 (1.02,5.75); 0.044**	1.21 (0.79,1.85); 0.383	**2.38 (1.02,5.54); 0.045**
Age (years)							
<55	50/70	29/41	7/4	0.99 (0.54,1.80); 0.974	2.45 (0.68,8.82); 0.170	1.12 (0.63,1.98); 0.696	2.46 (0.70,8.69); 0.162
≥55	90/130	64/71	14/10	1.30 (0.85,2.01); 0.231	2.02 (0.86,4.75); 0.106	1.39 (0.92,2.10); 0.116	1.83 (0.79,4.23); 0.159
Smoking							
Nonsmoker	76/108	38/63	9/5	0.86 (0.52,1.41); 0.544	2.56 (0.83,7.93); 0.104	0.98 (0.61,1.58); 0.941	2.70 (0.88,8.26); 0.082
Smoker	64/92	55/49	12/9	1.61 (0.98,2.66); 0.061	1.92 (0.76,4.82); 0.166	**1.66 (1.03,2.67); 0.036**	1.58 (0.64,3.88); 0.319
Alcoholism							
No	70/99	49/54	9/9	1.28 (0.78,2.10); 0.322	1.41 (0.53,3.74); 0.485	1.30 (0.81,2.08); 0.271	1.29 (0.50,3.34); 0.606
Yes	70/101	44/58	12/5	1.10 (0.67,1.80); 0.721	**3.46 (1.17,10.27);0.025**	1.28 (0.80,2.06); 0.301	3.35 (1.15,9.76); 0.027
BMI							
<25	62/80	39/41	6/6	1.23 (0.71,2.13); 0.465	1.29 (0.40,4.20); 0.672	1.24 (0.73,2.09); 0.431	1.20 (0.38,3.83); 0.761
≥25	78/120	54/71	15/8	1.17 (0.74,1.84); 0.498	**2.89 (1.17,7.13); 0.022**	1.34 (0.87,2.07); 0.179	**2.71 (1.12,6.58); 0.027**

Bold values are statistically significant (*P* <0.05).

## Discussion

In the present study, we evaluated the association between MGP gene rs1800802 polymorphism and the risk of knee OA in a Chinese population and found that rs1800802 polymorphism increased the risk of knee OA. Subgroup analyses obtained similar findings among the females, smokers, drinkers, and BMI ≥25 kg/m^2^ groups.

MGP, a kind of secreted protein, acts as an inhibitor of bone morphogenetic protein signaling and has high affinity to calcium ions [13]. Luo et al. [7] found that the growth plate showed inappropriate cartilage calcification in MGP-deficient mice. El-Maadawy et al. [8] suggested that the absence of MGP resulted in an accelerated calcification of cartilage. In addition, lower expression of MGP may lead to the development of osteochondrosis as a result of destitute blood and nutrient for the cartilage [9]. Those abovementioned animal experiments suggested that MGP may be related to the development of OA.

Misra et al. [11] first found MGP gene rs1800802 polymorphism was associated with decreased risk for hand OA. However, no significant association was found between serum MGP levels and radiographic hand OA [11]. In a recent study, no significant associations were observed between MGP rs1800802 polymorphism and the knee OA risk in a Mexican population [12]. In the present study, G allele or GG genotype of rs1800802 polymorphism in MGP gene was found to increase the risk of knee OA. It is obvious that the findings of this study were different from those of abovementioned studies. The following factors may explain them. First, the ages of OA patients were remarkably younger in our study (58.39 years old), while Misra et al. [11] enrolled OA patients with mean age 74 years. Second, the types of OA were diverse among these studies. Another factor may be the distinction of disease severity. The influence of different BMI, smoking habit, comorbidity like diabetes, may also contribute to those inconsistent findings. To our knowledge, this is the first study to explore the association between MGP gene rs1800802 polymorphism and knee OA risk in a Chinese population. Subgroup analyses observed positive findings in the female, smoker, drinker, and BMI ≥25 kg/m^2^ groups, suggesting that OA patients were more likely to expose to these factors.

Several potential limitations of this study should be considered. First, the relationship between the MGP gene polymorphism and knee OA susceptibility could not be fully revealed by a single case–control study because of the relatively small sample and it might underpower the facticity. Second, the cases and volunteers were selected from hospitals, which may not exactly represent the general population. Third, we did not obtain detailed information about severity of OA and its response to treatment respectively, which restricted our further analyses of the role MGP gene polymorphism played in OA. Fourth, we only investigated one SNP of MGP gene. Fifth, we did not know how the gene variant of MGP affects the development of knee OA. Sixth, the interacting between MGP gene polymorphisms and other environmental factors was not explored. Last but not the least, like other studies, we only evaluated the effects of MGP gene polymorphism among Chinese population, which may not generalize to other ethnic groups; so further studies with larger sample sizes in other races are warranted to confirm our findings.

To sum up, this study shows that MGP gene rs1800802 polymorphism may contribute to the increased risk for knee OA. However, this is only a preliminary conclusion as our results were obtained in Chinese Han population. Better designed and more multicenter case–control studies from diverse ethnic populations are needed to validate our findings.

## Abbreviations

BMI: body mass index
CI: confidence interval
dNTP: deoxy-ribonucleoside triposphate
HWE: Hardy–Weinberg equilibrium
K–L: Kellgren–Lawrence
MGP: matrix Gla protein
OR: odds ratio
RLFP: restriction fragment length polymorphism
